# Primates’ behavioural responses to tourists: evidence for a trade-off between potential risks and benefits

**DOI:** 10.1038/srep32465

**Published:** 2016-09-15

**Authors:** Laëtitia Maréchal, Ann MacLarnon, Bonaventura Majolo, Stuart Semple

**Affiliations:** 1 University of Roehampton, Department of Life Sciences, London SW15 4JD, UK; 2 University of Lincoln, School of Psychology, Lincoln LN6 7TS, UK

## Abstract

The presence of, and interactions with tourists can be both risky and beneficial for wild animals. In wildlife tourism settings, animals often experience elevated rates of aggression from conspecifics, and they may also be threatened or physically aggressed by the tourists themselves. However, tourist provisioning of wild animals provides them with highly desirable foods. In situations of conflicting motivations such as this, animals would be expected to respond using behavioural coping mechanisms. In the present study, we investigated how animals respond to tourist pressure, using wild adult Barbary macaques in the Middle Atlas Mountains, Morocco, as a case study. We found evidence that these animals use a range of different behavioural coping mechanisms–physical avoidance, social support, affiliative, aggressive and displacement behaviours–to cope with the stress associated with tourists. The pattern of use of such behaviours appears to depend on a trade-off between perceived risks and potential benefits. We propose a framework to describe how animals respond to conflicting motivational situations, such as the presence of tourists, that present simultaneously risks and benefits.

The simple presence of tourists close to wildlife, as well as the interactions between them, may be perceived as stressful by the animals involved^1^,^2^,^3^. Tourism is often associated with changes in animals’ natural behavioural patterns, and in particular increases in levels of conspecific aggression^4^,^5^,^6^,^7^. Tourists may also pose an aggressive threat–whether intentionally or not-towards animals, for example by crowding them^8^,^9^, waving objects at them, or in extreme cases chasing or physically attacking them^2^. It has been argued that in such contexts, tourists may be perceived similarly to natural predators in terms of the risks they pose; this perceived risk in turn promotes anti-predator behaviours, such as avoidance, to cope with the stressor^10^,^11^. However, tourists also often provide animals with highly attractive food^12^. As a result, animals may perceive tourists as motivationally conflicting stimuli; tourists may offer animals the opportunity to gain food, whilst at the same time posing a potential risk. In such conflicting situations, animals might respond to tourists using a range of behavioural coping mechanisms (defined as responses to aversive situations^13^), in order to increase benefits (i.e. acquiring food), while reducing the potential risks and/or managing the associated stress.

Animals are able to use a range of behavioural coping mechanisms to respond to stressful situations. A well-known coping mechanism is avoidance behaviour, where animals maintain an appropriate distance from a stressor^11^, or position themselves such that they can make a rapid and effective escape from the threat^14^. For example, potential prey flee when they perceive a predator^10^,^11^,^15^ or stay close to a potential escape route when predation risk is high^14^. Proximity to overhead tree cover may reduce the distance at which an animal flees from a potential threat; this effect has been demonstrated for ring tailed lemurs, *Lemur catta*^16^ and koomal, *Trichosurus vulpecula hypoleucus*^17^. In a number of taxa, including black howler monkeys, *Alouatta caraya*^18^, pygmy marmosets, *Callithrix pygmaea*^19^, urban birds^10^, and bottlenose dolphins, *Tursiops truncatus*^20^, animals have been seen to use avoidance behaviours when tourist number increases.

Social behaviours can also act as coping mechanisms. For example, in a wide variety of species the presence of a socially bonded partner has been shown to help animals cope better with stress, a phenomenon known as “social buffering”^21^,^22^. For instance, birds were found to be less tolerant to human disturbance, and thus they were more likely to flee their nest, when the number of conspecifics nearby was lower^23^. In Tibetan macaques, *Macaca thibetana*, it was suggested that the increased proximity to conspecifics observed when tourists were present may help these animals to cope with tourist pressure; however, such proximity may also be related to increased tolerance between animals when provisioning is taking place, rather than reflecting a coping strategy^24^. Affiliative behaviours directed toward or received by conspecifics have also been proposed to act as a coping mechanism^25^. During provisioning, increases in affiliative behaviour are likely to result from increased intraspecific competition, and to be used by animals to reduce social tension^26^. Most findings on social behaviours as coping mechanisms, particularly in non-human primates, are based on quantifying affiliative behaviour through grooming frequency. However, grooming may not be an appropriate measure of coping behaviour in a tourist context as provisioning might disrupt this activity, as has been found in male Barbary macaques^7^ and female bonnet macaques, *Macaca radiata*^27^. The potential role in coping of other affiliative behaviours such as short-term affiliative behaviours (non-aggressive social behaviour lasting a few seconds e.g. teeth chattering or embracing) has rarely been explored, and only in non-human primates. Where it has, the results present an inconsistent picture: short-term affiliative behaviour rates decreased during provisioning compared to foraging on natural items in female bonnet macaques^27^, while in male Barbary macaques, short-term affiliative behaviour rates increased when tourists were in close proximity, a situation in which they often provide food^28^.

Directing aggression toward other individuals can also act as a coping behaviour in stressful situations^29^. The rate of conspecific aggression has been shown to increase when animals are close to tourists, especially when food is provided, in southern stingrays, *Dasyatis americana*^6^, sicklefin lemon sharks, *Negaprion acutidens*^30^, Japanese and rhesus macaques, *Macaca fuscata* and *Macaca mulatta*^31^. However, it is difficult to determine whether this increase in aggression reflects the use of agonism as a coping mechanism, or rather is due to high intraspecific competition over supplementary food.

Displacement behaviours, defined as behaviours with an apparent lack of relevance to the context in which they occur, have also been proposed to act as coping mechanisms^32^,^33^,^34^. These behaviours include self-directed behaviours such as self-scratching and self-grooming^34^,^35^, plus other behaviours such as restlessness, which is defined as frequent changes in an individual’s behaviour^36^. Increased rates of displacement activities when in the presence of tourists have been documented, for example, in royal penguins, *Eudyptes schlegeli*^1^, and mountain gorillas, *Gorilla beringei beringei*^37^. In male Barbary macaques, rates of self-scratching increased when the mean number of tourists present increased or when animals interacted with tourists; such rates were not, however, related to other tourist pressure measures such as the maximum number of tourists present, or the closest proximity of tourists to the macaques^2^. These findings suggest that animals may use displacement behaviours to cope with some aspects of tourist pressure, but not others^2^.

Wildlife tourism is often promoted as a useful tool for the conservation of endangered species^4^,^38^. Assessing how animals respond to tourists is crucial if such tourism is to be well managed and sustainable^4^. In particular, it is important to consider the wide range of possible behavioural coping mechanisms that animals might employ in response to tourists, and how the different aspects of tourism–e.g. tourist number or behaviour–might affect animals. In this study, we investigated how wild adult Barbary macaques respond to tourist pressure. We considered a range of behavioural responses, testing whether Barbary macaques cope with the stress associated with tourists by using: avoidance behaviours, i.e. being off the ground, being under tree cover or being further away from tourists on the ground (Hypothesis 1); support from a socially bonded partner, defined as the three same-sex conspecifics that spent the most time in proximity and grooming with the focal macaque (Hypothesis 2); affiliative social behaviours (Hypothesis 3); aggression toward subordinate conspecifics (Hypothesis 4); or displacement behaviours, i.e. self-scratching and restlessness (Hypothesis 5). The findings of this study add to our understanding of the potential impacts of wildlife tourism, and have relevance to other contexts where primates–or other taxa–are visited by tourists. More broadly, investigating such issues can provide insights into how animals respond to situations that present both potential risks and benefits, and hence are motivationally conflicting.

## Results

Group scans were recorded every 30 min between 8 am to 5 pm. Tourists were present in 67.9% of the overall scans and in 99.8% of the scans recorded at the tourist site, the area where tourists encounter the macaques, with tourist numbers ranging from 1 to over 200 at any given time. Tourist-macaque interactions (TMI) occurred in 15.3% of the scans when tourist were present, with 90.5% of these interactions occurring when tourists were within 5 m distance from macaques on the ground. ‘Other’, non-physical interactions, such as taking pictures or waving at the macaques accounted for 55.6% of the total interactions, followed by feeding interactions (38.6%), and agonistic interactions (5.7%). In 83.1% of the interactions, up to 5 tourists were involved.

We used generalised linear mixed models (GLMM) to test the following five hypotheses (see method section for details). For each hypothesis, we first ran a series of GLMMs to explore whether avoidance behaviour (Hypothesis 1), support from a socially bonded partner (Hypothesis 2), affiliative social behaviours (Hypothesis 3), aggression toward subordinate conspecifics (Hypothesis 4) or displacement behaviours (Hypothesis 5) were predicted by the number of tourists in the area and in the nearest tourist group, or the occurrence of TMI (Table 1). We then conducted a second series of GLMMs to investigate whether each behavioural response was predicted by the different types of TMI (Table 2).

### Hypothesis 1. Barbary macaques use avoidance behaviours to cope with tourist pressure

Contrary to the predictions of this hypothesis, macaques did not appear to use avoidance behaviours when interacting with tourists and, in fact, opposite patterns to those expected were seen: during TMIs, macaques were more likely to be on the ground than off the ground, more likely to be in open space than under tree cover, and in closer proximity to tourists (Table 1). However, when comparing the different types of TMI, macaques seemed to use different avoidance behaviours depending on the type of TMI (Table 2). Macaques were more likely to be off the ground during ‘other’ interactions compared to feeding and agonistic interactions, and during agonistic interactions compared to feeding interactions. They were also more likely to be found in open space during feeding interactions compared to ‘other’ interactions, but there was no significant difference between agonistic interactions and the two other types of TMI. Macaques appeared to be further away from tourists during agonistic and ‘other’ interactions compared to feeding interactions.

There was support for Hypothesis 1 in relation to another aspect of tourist pressure: Barbary macaques were more likely to be off the ground or under tree cover when more tourists were present in the area; no such relationship was seen with the number of tourists in the nearest tourist group (Table 1). However, when macaques were on the ground, they were closer to-not, as expected, further away from-tourists when there was a larger number of tourists in the area and in the nearest tourist group (Table 1).

### Hypothesis 2. Barbary macaques use social support to cope with tourist pressure

In support of this hypothesis, Barbary macaques were more likely to be within 5 m of a closely bonded partner during tourist-macaque interactions compared to when no interaction occurred (Table 1). Such presence of a partner was not, however, related to the number of tourists in the area or in the nearest tourist group. The probability of a socially bonded partner being present did not differ significantly across the different types of tourist-macaque interactions (Table 2).

### Hypothesis 3. Barbary macaques use affiliative social behaviours to cope with tourist pressure

Rates of short-term affiliative social behaviour were higher during than outside of tourist-macaque interactions, supporting this hypothesis (Table 1), and these rates were more likely to be higher during agonistic and feeding interactions compared to ‘other’ non-physical interactions (Table 2). In contrast to expectations, however, rates of such affiliative behaviour were lower when there were more tourists in the area, or in the nearest tourist group (Table 1).

### Hypothesis 4. Barbary macaques use aggression to cope with tourist pressure

As predicted, rates of aggression to subordinates were higher during tourist-macaque interactions than when such interactions did not occur (Table 1); these rates were more likely to be higher during agonistic and feeding interactions than during ‘other’ non-physical interactions (Table 2). Aggression rates were unrelated to the number of tourists in the area or in the nearest tourist group (Table 2).

### Hypothesis 5. Barbary macaques use displacement behaviour to cope with tourist pressure

In support of this final hypothesis, rates of self-scratching and restlessness were higher during tourist-macaque interactions than when no interaction occurred (Table 1). Rates of self-scratching were not related to the number of tourists in the area or in the nearest group. Contrary to predictions, restlessness decreased when the number of tourists in the nearest tourist group increased; restlessness was unrelated to the number of tourists in the area. Macaques were more likely to have higher restlessness during feeding or agonistic interactions compared to ‘other’ non-physical interactions with tourists (Table 2); no effect of interaction type was seen for self-scratching.

## Discussion

For wild animals, tourist encounters and interactions potentially represent risks, which can be direct (e.g. aggression from tourists) or indirect (e.g. increased intraspecific competition over tourist-provisioned food)^2^,^31^,^39^. However, wildlife tourists can also bring highly attractive food, and therefore animals might perceive potential benefits from this situation^12^. The conflicting motivations arising from such situations may necessitate the use of coping strategies. The present study provided evidence that Barbary macaques use a range of behavioural coping mechanisms-avoidance, social support, affiliative, aggressive and displacement behaviours-to cope with tourists. Moreover, these mechanisms appeared to be used in response to specific aspects of tourist pressure, possibly reflecting a trade-off that animals make in each situation between the perceived risks and benefits associated with tourists.

The results indicate that Barbary macaques used two avoidance strategies-being off the ground or under tree cover-when very high numbers of tourists were present at the site. These are typical anti-predator strategies, and their apparent use here to cope with tourists is in line with findings in seabirds^40^, marmosets^19^, dolphins^41^, and Tibetan macaques^23^. However, when macaques were on the ground, they were more likely to be in close proximity to tourists when the latter’s number increased (and hence, presumably, so did food opportunities). Moreover, avoidance behaviours were less likely to occur when macaques interacted with tourists than when no interaction was taking place. This indicates that animals were highly attracted to tourists or allowed tourists to come in close proximity. However, macaques seemed to use different avoidance behaviours, according to the type of interaction with tourists. For instance, macaques were more likely to be off the ground or further away from tourists during agonistic interactions compared to feeding interactions. Together, these results suggest that study animals adjusted their avoidance behaviours to balance the potential risks perceived from high tourist numbers or agonistic interactions with tourists against the benefits related to the food provided by tourists.

Barbary macaques at the study site appeared to seek social support specifically to cope with the stress associated with interacting with tourists: an animal was more likely to have a socially bonded partner in close proximity during such interactions than outside of these events. It was not possible to exclude the possibility that such spatial patterns arose due to provisioning bringing animals into close proximity but, interestingly, the presence of a socially bonded partner was equally likely during ‘other’ non-physical interactions as during those that involved feeding and/or aggression from tourists. Recent evidence indicates that social buffering is important in (non-tourist exposed) wild Barbary macaques: adult males with stronger social bonds showed attenuated physiological responses to stressors^42^. In a tourism context, social support provided by a partner’s presence may help buffer animals against the high levels of anxiety they experience during close encounters with people^2^.

Short-term affiliative behaviour also appeared to offer Barbary macaques a way to cope with the stress associated with tourists. Levels of such behaviour increased when macaques interacted with tourists, with higher rates occurring during agonistic and feeding interactions, compared to ‘other’ non-physical interactions. The rates of these brief affiliative interactions were not, by contrast, positively related to tourist numbers and were in fact lower when the number of tourists increased in the area and in the nearest tourist group. Thus, an increase in short-term affiliative behaviour might serve to reduce social tension during provisioning^26^,^43^. These behaviours also appear to help animals cope specifically with the stress associated with tourist aggression, intraspecific competition during provisioning or the close proximity to tourists that such feeding elicits rather than the presence of tourists *per se*; as such, they appear to play a role in coping with highly stressful situations.

Aggression towards subordinate conspecifics may have a similar coping role to that of short-term affiliative behaviour: aggressive behaviour was higher when tourist-macaque interactions occurred than when there was no interaction, and higher rates of aggressive behaviour occurred during agonistic and feeding interactions compared to ‘other’ non-physical interactions. No relationship was seen with tourist numbers. From the point of view of the well-being of the animals at the study site, such elevated levels of aggression raise concerns, as they may be linked with increased physiological stress levels or risks of injury in the animals being aggressed^5^,^6^; regulating tourist-macaque interactions would therefore reduce these potential costs for animal welfare.

Displacement behaviours, finally, appeared to help Barbary macaques cope with stress associated with interacting with tourists, but patterns of results differed for restlessness and scratching: animals were more restless during feeding and agonistic interactions with tourists compared to ‘other’ non-physical interactions, but interaction type did not seem to affect self-scratching rates. These results support previous findings concerning self-scratching in male Barbary macaques^2^, suggesting that interacting with tourists increase anxiety, and such anxiety is related to the mere presence of tourists and not only associated with the increased intraspecific competition during provisioning. By contrast, observed increases in restlessness may more be related to such competition than to the presence of tourists *per se.* This is suggested because restlessness was lower, not higher, when the number of tourists in the nearest tourist group increased. In many studies of animals’ responses to stressors, particularly in primates, scratching or self-directed behaviours are the only measure of displacement behaviour examined^44^,^45^; the current study highlights the value of considering a range of displacement behaviours and of exploring the potential coping role of each of these separately.

**Figure 1:**
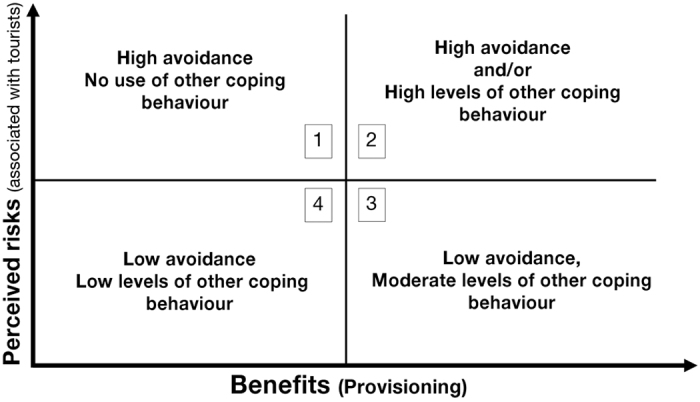
Framework for the trade-off for animals between the perceived risks and benefits related to tourists. In quadrant 1, animals perceive high risks associated with tourists but there is little attraction due to little or no provisioning occurring. In quadrant 2, animals still perceive high risks from tourists but the link between human presence and food presents significant potential benefits. In quadrant 3, the risk is perceived as low and is exceeded by a strong attraction for food. Finally, in quadrant 4, both the risks and the benefits related to tourists are perceived as low.

Overall, the results presented here indicate that Barbary macaques’ responses to tourism vary according to different aspects of tourist pressure, and that animals make a trade-off between potential risks and benefits associated with tourists, adjusting their behavioural responses accordingly. Therefore, based on the risk-disturbance hypothesis, which suggests that animals cope with human disturbance by making a trade-off directly related to energy gain^15^, we propose a framework for assessing how animals adapt their use of coping mechanisms, depending on the trade-off between the perceived risks and benefits (Fig. 1). This framework relates to how animals are expected to react to tourists; the benefits and costs considered are those that are short-term, and do not take into account the direct risks of tourist provisioning for animal health, as described for example in Maréchal *et al*.^3^.

In quadrant 1 of Fig. 1, animals perceive high risks associated with tourists but there is little attraction due to little or no provisioning occurring; here it is predicted that animals will use an active avoidance strategy, keeping a large distance from tourists, being off the ground or under tree cover. This strategy was observed in the present study when a large number of tourists were present at the tourist site; in such situations, macaques were more likely to be off the ground. In quadrant 2, animals still perceive high risks from tourists but the link between human presence and food presents significant potential benefits. Here, animals may modulate their flight distance according to the intensity of the perceived risk such that high rates of avoidance may occur, but where attraction is high enough, high levels of other coping behaviours (seeking social support, short-term affiliative social behaviours, aggression toward subordinate conspecifics or displacement behaviours) serve to alleviate the associated stress. In the present study, macaques were more likely to be off the ground and had higher rates of restlessness or affiliative behaviours during agonistic and feeding interactions compared to ‘other’ non-physical interactions. In quadrant 3, the risk is perceived as low and is exceeded by a strong attraction for food; as a result, animals may not avoid tourists, and they may cope with any residual emotional conflict between risks and benefits through moderate levels of other coping behaviours. For example, macaques in the present study were more attracted to tourists during feeding interactions compared to any other interactions, but presented high rates of displacement behaviours. Finally, in quadrant 4, if both the risks and the benefits related to tourists are perceived as low, for example when the number of tourists is low and natural food availability is high, animals may show low levels of both avoidance or other coping; this configuration of low risks and benefits would potentially reduce the impacts of tourism on animal welfare and is predicted to occur when animals are highly habituated but provisioning is regulated or absent.

Clearly, the perceived risk from tourists is likely to be affected by the interplay between animals’ individual characteristics (e.g. species, age, sex, degree of habituation, temperament, previous experience), and the context (i.e. tourist number, tourist and conspecific behaviour). The perceived benefit is also dependent on a multitude of factors, for example food preference, amount of food previously consumed and nutritional status. More data are now needed to parameterise the framework. In addition, its use might be extended to assess the range of behavioural responses associated with the trade-off between perceived risks and benefits within an individual at different times, to compare between individuals, or more broadly to compare species. The framework may also be used to compare and assess different tourism disturbance and settings. For example, the framework could be used to assess animal welfare, and facilitate management decisions on how to improve wildlife tourism, by determining which quadrant best represents animals’ responses to tourists under different tourism contexts. Finally and more generally, the framework provides a potentially useful tool for understanding animals’ behavioural coping mechanisms in contexts of motivational conflict; as it considers generic (i.e. not taxon-specific) avoidance and other coping behaviours, we feel it should be relevant and applicable to a wide range of vertebrate taxa.

## Methods

### Study sites and animals

The Barbary macaque is an endangered species, native to Morocco and Algeria; in the former country, tourism has been proposed as a potential tool for its conservation^46^. Data were collected on one group of Barbary macaques located in Ifrane National Park in the Middle Atlas Mountains, Morocco (33°25.0N; 005°10.0W). This group experiences high daily tourist visitation rates and provisioning is common; tourists have been visiting monkeys at this site for over ten years^2^. At the start of the data collection, the group was composed of 40 individuals: 12 adult males, 12 adult females, 2 sub-adult males, 1 sub-adult female, 6 juveniles, 7 one-year-old infants, and 5 infants were born during the study period. Data were collected on 17 adult individuals (8 males and 9 females). One adult male and one adult female were excluded from data collection as they died early in this process. Young adults (3 males, 2 females) were also excluded from data collection due to time constraints.

### Data collection

This work followed the Animal Behaviour Society’s guidelines for the treatment of animals in behavioural research and teaching, adhered to standards as defined by the European Union Council Directive172 86/609/EEC, and was approved by the Ethics Committee of the University of Roehampton (LSC 15/124). The study was conducted from 9th February to the 29th December 2012, with a total of 207 days of data collection, during which the group was monitored from 8am to 5pm. Behavioural data were collected by LM and three to four field assistants at any one time; regular inter-observer reliability tests were conducted. All data were collected using Psion handheld computers loaded with Pendragon Forms Version 5.1 (©Pendragon Software Cooperation, USA).

Two types of behavioural sampling^47^ were used to assess macaques’ behavioural responses to tourists. Using scan sampling, data on avoidance behaviours were recorded every thirty minutes. Avoidance behaviours comprised three different measures: being off the ground (e.g. in a tree), being under tree cover, and the distance from the nearest tourist group when on the ground. Tourist measures recorded during scan samples were the number of tourists present in the area (i.e. within 100 m of the core of the macaque group^2^), and the number of tourists in the nearest tourist group to each macaque (a group was defined as an aggregation of tourists within 3 m of each other). A maximum of 19 scans (mean = 8; s.d. = 3.4) was recorded per individual per day.

Continuous focal sampling was used to assess social and displacement behaviours. A total of 625.5 hours of focal observation (mean = 36.8 hrs, ranging from 31.5 hrs to 39.0 hrs per individual) was recorded. Social variables recorded were: the presence of a closely socially bonded partner (see below for definition) within 5 m of the focal macaque, short-term affiliative behaviours excluding grooming, (i.e. teeth chattering, same-sex genital inspection, triadic interaction with infant, embrace, non-aggressively grabbing hindquarter of a conspecific), and the giving of aggressive behaviours toward subordinate conspecifics. Two displacement behaviours were recorded: self-scratching and restlessness (rate of change in activity behaviours). Each study animal, randomly selected, was followed for 10 minutes, once a day, on each study day. Aggressive behaviours, self-scratching and short-term affiliative behaviours were recorded as frequencies; two events were distinguished when they were separated by at least 10 seconds. During focal watches, durations of mutually exclusive activities–grooming, resting, travelling, feeding and vigilance–were recorded; this allowed calculation of restlessness (the rate of change between these activities) and also gave data on grooming that were used to calculate a ‘composite index of sociality’ score (see below). For each study animal, their socially bonded partners were defined as the three same-sex conspecifics that spent the most time in proximity and grooming with them, as determined by the ‘composite index of sociality’ (CSI^48^). When tourist-macaque interactions (TMIs, see below) started, when self-scratching occurred and during feeding activity on natural or human food, the presence or absence within 5 m of all other adults was noted, and these data later assessed to check if any of these animals was a socially bonded partner.

A tourist-macaque interaction was defined as any behavioural exchange between one or several tourists and a macaque. TMIs were categorised into three types as described in Maréchal *et al*.^2^: (a) Agonistic interactions, in which tourists chased a macaque, threw or pretended to throw non-food items towards them, or where they physically struck or pushed them. (b) Feeding interactions in which tourists handed food items to a macaque or threw such items towards them. (c) ‘Other’, non-physical interactions (called ‘neutral interactions’ in Maréchal *et al*.^2^) in which tourists engaged with but made no attempt to interact physically with a macaque, namely where tourists talked to, waved at or photographed the animal, or were within 2 m of them. As several types of interactions could occur during a focal sampling observation, the TMI type for each focal observation was classified hierarchically. When an agonistic interaction occurred, even if feeding and ‘other’ non-physical interactions occurred in the same focal observation, the interaction was classified as agonistic. When a feeding interaction occurred without any agonistic interaction but together with ‘other’ non-physical interactions, the interaction was classified as a feeding interaction. An interaction was classified as ‘other’ non-physical interaction when only this type of interaction occurred. A TMI was considered to have ended when no subsequent interaction between tourists and the focal macaque occurred for ≥30 seconds and the tourists were at a distance of more than 2 metres from the focal animal.

In order to investigate whether the study animals had higher social or displacement behaviours during TMIs, a post-TMI/matched control comparison was used (similar to the post-conflict/matched control method^49^). When a TMI between the focal macaque and tourists ended, an additional 10 min of continuous focal sampling data was collected (post-TMI). Matched control (MC) observational data for the same animal were collected within one week, before or after the matched post-TMI focal, at a similar time of day and when the macaque had had no TMI within 10 min prior to or during the observation^49^.

Individual dominance rank, social season and average daily temperature were recorded and used as control variables in the analytical models, as these variables might influence animals’ behavioural responses to tourists. To determine individual rank, *ad libitum* sampling was used to record the outcomes of all visible same-sex dyadic conflicts with no counter-aggression, and used to calculate the dominance rank of each study animal using corrected normalized David’ scores^50^. Three social seasons were determined (i.e. birth, mating and ‘other’). Birth season lasted from the first birth to the birth of the last infant of the year, i.e. from 6^th^ April to 1^st^ May 2012. Mating season lasted from the first to the last ejaculatory copulations observed (following the definition in Young *et al*.^42^), i.e. from 9^th^ February to 6^th^ March 2012, and from 15^th^ September to 29^th^ December 2012. ‘Other’ was the period between birth and mating seasons. The temperature was recorded every hour during the study day, using a Kestrel 3500 Pocket Weather Meter.

### Statistical analysis

To test the five hypotheses, a series of GLMMs^51^ was used to explore the relationship between tourist variables and Barbary macaques’ use of avoidance, social and/or displacement behaviours. The dependent, independent and control variables, and random factors included in the models are listed in Tables 3 and 4.

Two types of models were conducted to assess which tourist variables predicted the use of avoidance behaviour, social support, affiliative, aggressive and displacement behaviour. The first type of model explored the relationships between each of these and different measures of tourist pressure, namely the number of tourists in the area and in the nearest tourist group, and occurrence of TMI (models: 1.1a, 1.2a, 1.3a, 2a, 3a, 4a, 5.1a, 5.2a-Table 3). The second type of model was run to determine which type of TMI (i.e. agonistic, feeding or ‘other’ interactions; see details above) predicted avoidance behaviours, and higher behavioural responses during TMIs compared to matched controls, including social support, affiliative, aggressive and displacement behaviours (models: 1.1b, 1.2b, 1.3b, 2b, 3b, 4b, 5.1b, 5.2b–Table 4).

All models were fitted using R 3.1.3^52^. The models including as the dependent variables distance between tourists and macaques on the ground, and the rates of social and displacement behaviours used the function lme of the R-package nlme for Gaussian linear mixed-effects models^53^. The significance of the individual fixed effects was determined based on the t- and p-values provided by lme^54^. All other models used the function glmer of the R-package lme4, family = ”binomial” for binomial mixed-effects models^55^. The significance of the individual fixed effects was determined based on the z- and p-values provided by lmer^55^.

For each model, the significance of the full final model was compared to the corresponding null model using a likelihood ratio test (R function ANOVA)^56^. The null model corresponds to the full model excluding all independent variables, which are replaced by the value 1 in the model. The full model was used to test the effects of individual predictors and not the “best fit model”, as recommended by Mundry and Nunn^57^. All models were checked to assess whether they violated any assumptions. Collinearity between independent variables was checked using variance inflation factors^58^. The VIF function of the R-package “car” for the Gaussian models^59^ and the vif.mer function of the R-package “nlme”^54^ were applied to the full linear model excluding the random effects. All VIF values were lower than 4, indicating low collinearity between predictors^58^; therefore, all predictors were included in the models. Furthermore, Cook’s distance was always lower than 1 in the dataset, indicating the absence of any outliers^58^. Categorical variables such as number of tourists in the area and in the nearest tourist group and rank were z-transformed to improve the interpretability of the variables^60^. For the Gaussian models, the assumptions for normal distribution and the homogeneity of the residuals were checked by visually inspecting a q-q plot where the residuals were plotted against fitted values^58^. When the assumption of normality was not met, the dependent variable was log-transformed or z-transformed in order improve normality in the distribution of the residuals. For the binomial models, the over dispersion of the data was also tested and accepted if the result was equal to 1.

## Additional Information

**How to cite this article**: Maréchal, L. *et al*. Primates' behavioural responses to tourists: evidence for a trade-off between potential risks and benefits. *Sci. Rep.* **6**, 32465; doi: 10.1038/srep32465 (2016).

## Supplementary Material

Supplementary Information

Supplementary Dataset 1

Supplementary Dataset 2

## Figures and Tables

**Table 1:** Results of the first series of GLMMs testing the relationships between the different potential coping behaviours and tourist pressure variables.

Hypothesis 1a: Avoidance behaviour					
1) *Being off the ground*	**Estimate**	**±SE**	**z**	***P***	**Direction**
Intercept	−0.625	0.110	−5.683	***<0.001***	
Total no. of tourists in the area	0.310	0.022	13.800	***<0.001***	**+**
No. of tourists in the nearest tourist group	0.009	0.020	0.437	0.663	
TMI (Yes vs. No)	−1.605	0.063	−25.522	***<0.001***	**−**
2) *Being under tree cover*	**Estimate**	** ± SE**	**z**	***P***	
Intercept	1.836	0.146	12.528	***<0.001***	
Total no. of tourists in the area	0.198	0.042	4.624	***<0.001***	**+**
No. of tourists in the nearest tourist group	0.020	0.036	0.555	0.579	
TMI (Yes vs. No)	−1.139	0.077	−14.671	***<0.001***	**−**
3) *Being further away from tourists*	**Estimate**	**±SE**	**t**	***P***	
Intercept	0.141	0.060	2.318	***0.002***	
Total no. of tourists in the area	−0.103	0.006	−15.748	***<0.001***	**−**
No. of tourists in the nearest tourist group	−0.019	0.001	−14.569	***<0.001***	**−**
TMI (Yes vs. No)	−1.689	0.014	−113.206	***<0.001***	**−**
Hypothesis 2a: Social support	**Estimate**	**±SE**	**z**	***P***	**Direction**
Intercept	−1.079	0.280	−3.855	***<0.001***	
Total no. of tourists in the area	−0.008	0.072	−0.112	0.911	
No. of tourists in the nearest tourist group	0.007	0.018	0.382	0.702	
TMI (Yes vs. No)	0.656	0.121	5.416	***<0.001***	**+**
Hypothesis 3a: Aggression	**Estimate**	**±SE**	**t**	***P***	**Direction**
Intercept	0.485	0.101	4.786	***<0.001***	
Total no. of tourists in the area	0.000	0.024	0.004	0.997	
No. of tourists in the nearest tourist group	−0.013	0.007	−1.761	0.078	
TMI (Yes vs. No)	0.95	0.049	19.535	***<0.001***	**+**
Hypothesis 4a: Affiliative behaviour	**Estimate**	**±SE**	**t**	***P***	**Direction**
Intercept	1.310	0.133	9.863	***<0.001***	
Total no. of tourists in the area	−0.079	0.028	−2.866	***0.004***	**−**
No. of tourists in the nearest tourist group	−0.016	0.008	−1.966	***0.049***	**−**
TMI (Yes vs. No)	0.543	0.055	9.846	***<0.001***	**+**
Hypothesis 5a: Displacement behaviour					
1) *Rates of self-scratching*	**Estimate**	**±SE**	**t**	***P***	**Direction**
Intercept	10.146	1.943	5.221	***<0.001***	
Total no. of tourists in the area	−0.116	0.332	−0.348	0.728	
No. of tourists in the nearest tourist group	−0.050	0.097	−0.518	0.605	
TMI (Yes vs. No)	4.923	0.662	7.434	***<0.001***	**+**
2) *Restlessness*	**Estimate**	**±SE**	**t**	***P***	**Direction**
Intercept	−0.334	0.090	−3.718	***<0.001***	
Total no. of tourists in the area	−0.034	0.022	−1.569	0.117	
No. of tourists in the nearest tourist group	−0.020	0.006	−3.139	***0.002***	**−**
TMI (Yes vs. No)	0.440	0.043	10.263	***<0.001***	**+**

P values in bold and italic are significant. The direction column indicates the direction of significant relationships. The full GLMM results can be found in supplementary Tables S1 and S3.

**Table 2:** Results of the second series of GLMMs testing the relationships between the different potential coping behaviours and the different types of tourist-macaque interactions.

	Estimate	±SE	z	*P*	Direction
Hypothesis 1b: Avoidance behaviour					
1) *Being off the ground*					
Intercept	−2.294	0.271	−8.453	***<0.001***	
Agonistic v. Feeding	−0.874	0.315	−2.777	***0.005***	**−**
Agonistic v. Other	1.531	0.001	1332.000	***<0.001***	**+**
Feeding v. Other	1.116	0.171	6.794	***<0.001***	**+**
2) *Being under tree cover*					
Intercept	4.247	0.922	4.602	***<0.001***	
Agonistic v. Feeding	−0.227	0.445	−0.509	0.610	
Agonistic v. Other	0.778	0.443	1.755	0.079	
Feeding v. Other	0.969	0.210	4.617	***<0.001***	**+**
3) *Being further away from tourists*					
Intercept	0.432	0.133	3.252	***0.001***	
Agonistic v. Feeding	−0.661	0.080	−8.235	***<0.001***	**−**
Agonistic v. Other	−0.090	0.079	−1.137	0.255	
Feeding v. Other	0.556	0.038	14.601	***<0.001***	**+**
Hypothesis 2b: Social support					
Intercept	−1.783	0.365	−4.884	***<0.001***	
Agonistic v. Feeding	0.093	0.216	0.431	0.666	
Agonistic v. Other	−0.127	0.273	−0.464	0.642	
Feeding v. Other	−0.219	0.276	−0.794	0.427	
Hypothesis 3b: Aggression					
Intercept	−1.220	0.332	−3.674	***<0.001***	
Agonistic v. Feeding	−0.279	0.173	−1.611	0.107	
Agonistic v. Other	−1.469	0.281	−5.227	***<0.001***	**−**
Feeding v. Other	−1.190	0.288	−4.140	***<0.001***	**−**
Hypothesis 4b: Affiliative behaviour					
Intercept	−2.124	0.513	−4.137	***<0.001***	
Agonistic v. Feeding	0.054	0.267	0.204	0.839	
Agonistic v. Other	−0.840	0.404	−2.080	***0.038***	**−**
Feeding v. Other	−0.885	0.411	−2.153	***0.031***	**−**
Hypothesis 5b: Displacement behaviour					
1) *Self-scratching*					
Intercept	−0.910	0.345	−2.635	***0.008***	
Agonistic v. Feeding	−0.224	0.171	−1.310	0.190	
Agonistic v. Other	−0.304	0.211	−1.443	0.149	
Feeding v. Other	−0.080	0.220	−0.365	0.715	
*2) Restlessness*					
Intercept	0.789	0.305	2.590	***0.010***	
Agonistic v. Feeding	0.155	0.173	0.898	0.369	
Agonistic v. Other	−1.043	0.198	−5.269	***<0.001***	**−**
Feeding v. Other	−1.199	0.208	−5.773	***<0.001***	**−**

P values in bold and italic are significant. The direction column indicates the direction of significant relationships; ‘-’ indicates a lower value for the second interaction type in the comparison, ‘+’ indicates a higher value for the second interaction type. The full GLMM results can be found in supplementary Tables S2 and S4.

**Table 3:** Variables included in the first series of GLMMs to test the relationships between Barbary macaques’ behavioural responses (dependent variables) and measures of tourist pressure (independent variables).

Hypothesis		1			2	3	4	5	
Model		1.1a	1.2a	1.3a	2a	3a	4a	5.1a	5.2a
		Dependent variable							
Independent variables		Macaques being off the ground (Yes vs. No)	Macaques being under tree cover (Yes vs. No)	Distance from tourists on the ground (z-transformed)	A socially bonded partner present within 5 m distance (Yes vs. No)	Rates of aggressive behaviours (log-transformed)	Rates of short-term affiliative behaviours (log-transformed)	Rates of self-scratching	Restlessness (z-transformed)
Tourist variables	Number of tourists in the area	✓	✓	✓	✓	✓	✓	✓	✓
	Number of tourists in the nearest tourist group	✓	✓	✓	✓	✓	✓	✓	✓
	TMI (Yes vs. No)	✓	✓	✓	✓	✓	✓	✓	✓
Control variables	Sex	✓	✓	✓	✓	✓	✓	✓	✓
	Rank	✓	✓	✓	✓	✓	✓	✓	✓
	Seasons (Birth vs. Mating vs. other periods)	✓	✓	✓	✓	✓	✓	✓	✓
	Daily temperature	✓	✓	✓	✓	✓	✓	✓	✓
	Random factors	macaque ID+Nested factors: Scan/Date			Date+macaque ID				

✓ Included in the model. TMI = Tourist Macaque Interaction. The letter ‘a’ corresponds to the first series of model conducted.

**Table 4:** Variables included in the second series of GLMMs to test the relationships between Barbary macaques’ behavioural responses (dependent variables) and the different types of tourist-macaque interactions (independent variables).

Hypothesis		1			2	3	4	5	
Model		1.1b	1.2b	1.3b	2b	3b	4b	5.1b	5.2b
Independent variables		Macaques being off the ground (Yes vs. No)	Macaques being under tree cover (Yes vs. No)	Distance from tourists on the ground (z-transformed)	A socially bonded partner more likely to be present within 5 m distance during TMI compared to MC	Higher rates of aggressive behaviours toward conspecifics during TMI compared to MC	Higher rates of short term affiliative behaviours during TMI compared to MC	Higher rates of self-scratching during TMI compared to MC	Higher restlessness during TMI compared to MC
Tourist variables	TMI (agonistic vs. feeding interactions)	✓	✓	✓	✓	✓	✓	✓	✓
	TMI (agonistic vs. ‘other’ interactions)	✓	✓	✓	✓	✓	✓	✓	✓
	TMI (feeding vs. ‘other’ interactions)	✓	✓	✓	✓	✓	✓	✓	✓
Control variables	Sex	✓	✓	✓	✓	✓	✓	✓	✓
	Rank	✓	✓	✓	✓	✓	✓	✓	✓
	Seasons (Birth vs. Mating vs. other periods)	✓	✓	✓	✓	✓	✓	✓	✓
	Daily temperature	✓	✓	✓	✓	✓	✓	✓	✓
	Random factors	macaque ID+Nested factors: Scan/Date			Date+macaque ID				

✓ Included in the model. TMI = Tourist Macaque Interaction. The letter ‘b’ corresponds to the second series of model conducted.
