# Supplementary material for: Primates’ behavioural responses to tourists: evidence for a trade-off between potential risks and benefits
Source: Sci Rep. 2016 Sep 15;6:32465. doi: 10.1038/srep32465 (PMC5032226; doi:10.1038/srep32465)
Supplement: Supplementary Information [file srep32465-s1.pdf]

Supplementary information

Primates' behavioural responses to tourists: evidence for a trade-off between potential risks and benefits.

Laëtitia Maréchal, Ann MacLarnon, Bonaventura Majolo, and Stuart Semple

Table S1: Results of GLMM testing the relationships between the different avoidance behaviours and tourist pressure variables. P values in bold and italic are significant.

Model Response	Null vs. Full model			Predictor variables									
				Intercept	Total n° of tourists in the area	N° of tourists in the nearest tourist group	TMI (Yes v. No)	Sex	Rank	Social season			Daily temp
										Birth vs Mating	Birth vs. Other	Mating vs. Other	
Being off the ground	N	19214	Estimate	-0.625	0.310	0.009	-1.605	-0.079	-0.221	0.150	-0.042	-0.187	-0.001
	df	7	± SE	0.110	0.022	0.020	0.063	0.107	0.045	0.087	0.091	0.056	0.003
	χ^2	1986.00	z	-5.683	13.800	0.437	-25.522	-0.743	-4.876	1.735	-0.458	-3.324	-0.153
	P	***<0.001***	*P value*	***<0.001***	***<0.001***	0.663	***<0.001***	0.458	***<0.001***	***0.083***	0.647	***<0.001***	0.878
Being under tree cover	N	19183	Estimate	1.837	0.199	0.020	-1.139	-0.207	-0.059	0.071	-0.572	-0.629	0.114
	df	7	± SE	0.147	0.043	0.036	0.078	0.079	0.031	0.140	0.145	0.099	0.006
	χ^2	0.00	z	12.528	4.624	0.555	-14.671	-2.634	-1.914	0.504	-3.945	-6.336	19.454
	P	***1.000***	*P value*	***<0.001***	***<0.001***	0.579	***<0.001***	***0.008***	0.056	0.614	***<0.001***	***<0.001***	***<0.001***
Being further away from tourists	N	12926	Estimate	0.317	-0.140	-0.089	-1.492	-0.036	0.040	0.286	0.071	-0.215	0.008
	df	7	± SE	0.046	0.008	0.007	0.016	0.012	0.006	0.068	0.071	0.045	0.003
	L.ratio	7637.00	t value	6.839	-18.103	-13.380	-90.882	-2.896	6.453	4.224	0.990	-4.728	3.182
	P	***<0.001***	*P value*	***<0.001***	***<0.001***	***<0.001***	***<0.001***	***0.004***	***<0.001***	***<0.001***	0.323	***<0.001***	***0.002***

Table S2: Results of GLMM testing the relationships between avoidance behaviours and the different types of TMI. P values in bold and italic are significant.

Model Response	Null vs. Full model		Predictor variables										
			Intercept	TMI			Sex	Rank	Social season			Daily temp
				Agg. Vs. Feed	Agg. Vs. Other	Feed. Vs. Other			Birth vs Mating	Birth vs. Other	Mating vs. Other	
Being off the ground	N	2991	Estimate	-2.294	-0.874	1.531	1.116	-0.455	0.045	1.221	0.414	-0.360	-0.027
	df	6	± SE	0.271	0.315	0.001	0.171	0.134	0.069	0.310	0.001	0.214	0.012
	χ^2	0.00	t value	-8.453	-2.777	1332.000	6.794	-3.390	0.655	3.935	360.000	-1.685	-2.215
	P	***1***	*P value*	***<0.001***	***0.005***	***<0.001***	***<0.001***	***<0.001***	0.512	***<0.001***	***<0.001***	0.091	***0.027***
Being under tree cover	N	2991	Estimate	4.247	-0.227	0.778	0.969	-0.786	0.005	0.353	-0.420	-1.647	0.151
	df	6	± SE	0.922	0.445	0.443	0.210	0.198	0.101	0.598	0.639	0.455	0.030
	χ^2	7.79	t value	4.602	-0.509	1.755	4.617	-3.976	0.051	0.591	-0.657	-3.623	5.017
	P	0.593	*P value*	***<0.001***	0.610	0.079	***<0.001***	***<0.001***	0.959	0.555	0.511	***<0.001***	***<0.001***
Being further away from tourists	N	2576	Estimate	0.432	-0.661	-0.090	0.556	-0.209	0.043	0.305	0.186	-0.092	-0.011
	df	6	± SE	0.133	0.080	0.079	0.038	0.036	0.018	0.117	0.121	0.083	0.005
	L ratio	298.21	t value	3.252	-8.235	-1.137	14.601	-5.827	2.384	2.623	1.532	-1.098	-2.324
	P	***<0.001***	*P value*	***0.001***	***<0.001***	0.255	***<0.001***	***<0.001***	***0.017***	***0.009***	0.127	0.274	***0.021***

Table S3: Results of GLMM testing the relationships between social support, affiliative, agonistic and displacement behaviours and tourist pressure variables. P values in bold and italic are significant.

	Model Response	Null vs. Full model			Predictor variables									
					Intercept	Total n° of tourists in the area	N° of tourists in the nearest tourist group	TMI (Yes vs. No)	Sex	Rank	Social season			Daily temp
											Birth vs Mating	Birth vs. Other	Mating vs. Other	
**Social behaviours**	Support presence (Yes vs. No)	N	955	Estimate	-1.079	-0.008	0.007	0.656	-0.414	-0.230	-1.483	-0.066	1.418	-0.012
		df	7	± SE	0.280	0.072	0.018	0.121	0.221	0.108	0.255	0.249	0.201	0.010
		χ^2	98.94	z value	-3.855	-0.112	0.382	5.416	-1.875	-2.110	-5.811	-0.263	7.044	-1.231
		P	***<0.001***	*P value*	***<0.001***	0.911	0.702	***<0.001***	0.061	0.035	***<0.001***	0.793	***<0.001***	0.218
	Rates of aggression towards conspecifics	N	2170	Estimate	0.485	0.000	-0.013	0.951	-0.041	-0.050	0.159	-0.133	-0.292	0.007
		df	8	± SE	0.101	0.024	0.007	0.049	0.066	0.033	0.090	0.095	0.062	0.004
		L.ratio	390.15	t value	4.786	0.004	-1.761	19.535	-0.615	-1.440	1.765	-1.394	-4.687	1.880
		P	***<0.001***	*P value*	***<0.001***	0.997	0.078	***<0.001***	0.548	0.149	0.078	0.163	***<0.001***	0.060
	Rates of affiliative behaviour	N	2170	Estimate	1.310	-0.079	-0.016	0.543	0.029	-0.120	-0.029	-0.218	-0.189	0.003
		df	8	± SE	0.133	0.028	0.008	0.055	0.123	0.058	0.102	0.108	0.071	0.004
		L.ratio	116.32	t value	9.863	-2.866	-1.966	9.846	0.238	-2.030	-0.283	-2.027	-2.680	0.816
		P	***<0.001***	*P value*	***<0.001***	***0.004***	**0.049**	***<0.001***	0.815	0.042	0.778	0.043	**0.007**	0.414
**Displacement behaviours**	Rates of self-scratching	N	2170	Estimate	10.146	-0.116	-0.050	4.923	5.706	1.493	-1.632	0.070	1.702	0.136
		df	8	± SE	1.943	0.332	0.097	0.662	2.194	0.967	1.228	1.293	0.850	0.048
		L.ratio	91.85	t value	5.221	-0.348	-0.518	7.434	2.601	1.545	-1.329	0.054	2.002	2.834
		P	***<0.001***	*P value*	***<0.001***	0.728	0.605	***<0.001***	**0.020**	0.123	0.184	0.957	**0.046**	**0.005**
	Rates of restlessness	N	2170	Estimate	-0.334	-0.034	-0.020	0.440	-0.131	-0.030	0.471	0.134	-0.337	0.003
		df	8	± SE	0.090	0.022	0.006	0.043	0.060	0.029	0.080	0.084	0.055	0.003
		L.ratio	167.99	t value	-3.718	-1.569	-3.139	10.263	-2.183	-0.940	5.926	1.601	-6.144	0.945
		P	***<0.001***	*P value*	***<0.001***	0.117	**0.002**	***<0.001***	0.045	0.347	***<0.001***	0.109	***<0.001***	0.345

Table S4: Results of GLMM testing the relationships between social support, affiliative, agonistic and displacement behaviours and the different types of TMI. P values in bold and italic are significant.

	Model Response	Null vs. Full model			Predictor variables									
					Intercept	TMI			Sex	Rank	Social season			Daily temp
						Agg. Vs. Feed	Agg. Vs. Other	Feed. Vs. Other			Birth vs Mating	Birth vs. Other	Mating vs. Other	
Social behaviours	Higher support presence during TMI than MC	N	955	Estimate	-1.783	0.093	-0.127	-0.219	-0.091	-0.105	-1.915	-0.602	1.309	0.029
		df	6	± SE	0.365	0.216	0.273	0.276	0.271	0.132	0.505	0.352	0.464	0.015
		χ^2	0.00	t value	-4.884	0.431	-0.464	-0.794	-0.336	-0.791	-3.792	-1.710	2.820	1.913
		P	***1***	*P value*	***<0.001***	0.666	0.642	0.427	0.737	0.429	***<0.001***	***0.087***	***<0.001***	***0.056***
	Higher aggression towards conspecifics during TMI than MC	N	955	Estimate	-1.220	-0.279	-1.469	-1.190	0.389	-0.247	0.231	-0.037	-0.268	0.008
		df	6	± SE	0.332	0.173	0.281	0.288	0.171	0.082	0.353	0.347	0.263	0.013
		χ^2	89.78	t value	-3.674	-1.611	-5.227	-4.140	2.270	-3.008	0.653	-0.107	-1.018	0.636
		P	***<0.001***	*P value*	***<0.001***	0.107	***<0.001***	***<0.001***	***0.023***	***<0.001***	0.513	0.915	0.309	0.524
	Higher affiliative behaviours during TMI than MC	N	955	Estimate	-2.124	0.054	-0.840	-0.885	1.217	-0.061	-0.499	-0.614	-0.114	-0.033
		df	6	± SE	0.513	0.267	0.404	0.411	0.300	0.139	0.504	0.502	0.386	0.022
		χ^2	0.00	t value	-4.137	0.204	-2.080	-2.153	4.057	-0.437	-0.988	-1.222	-0.297	-1.538
		P	***1.000***	*P value*	***<0.001***	0.839	***0.038***	***0.031***	***<0.001***	0.662	0.323	0.222	0.767	0.124
Displacement behaviours	Higher Self-scratching rates during TMI than MC	N	955	Estimate	-0.910	-0.224	-0.304	-0.080	0.484	0.037	-0.077	-0.031	0.046	-0.001
		df	6	± SE	0.345	0.171	0.211	0.220	0.224	0.106	0.351	0.336	0.256	0.013
		χ^2	61.57	t value	-2.635	-1.310	-1.443	-0.365	2.158	0.351	-0.220	-0.091	0.180	-0.096
		P	***<0.001***	*P value*	***0.008***	0.190	0.149	0.715	***0.031***	0.726	0.826	0.928	0.857	0.924
	Higher restlessness rates during TMI than MC	N	955	Estimate	0.789	0.155	-1.043	-1.199	0.055	-0.153	-0.739	-0.519	0.221	0.036
		df	6	± SE	0.305	0.173	0.198	0.208	0.161	0.075	0.324	0.310	0.238	0.012
		χ^2	113.53	t value	2.590	0.898	-5.269	-5.773	0.341	-2.053	-2.281	-1.674	0.927	3.083
		P	***<0.001***	*P value*	***0.010***	0.369	***<0.001***	***<0.001***	0.733	***0.040***	***0.023***	***0.094***	0.354	***<0.001***
